# Supplementary material for: Inhibition allocates spikes during hippocampal ripples
Source: Nat Commun. 2022 Mar 11;13:1280. doi: 10.1038/s41467-022-28890-9 (PMC8917132; doi:10.1038/s41467-022-28890-9)
Supplement: Supplementary file 1 — Supplementary Information [file 41467_2022_28890_MOESM1_ESM.pdf]

Supplementary Information for

Inhibition allocates spikes during hippocampal ripples

Asako Noguchi, Roman Huszár, Shota Morikawa, György Buzsáki, Yuji Ikegaya

Correspondence to: yuji@ikegaya.jp, Gyorgy.Buzsaki@nyulangone.org

This PDF file includes:

Supplementary Fig. 1 to 11

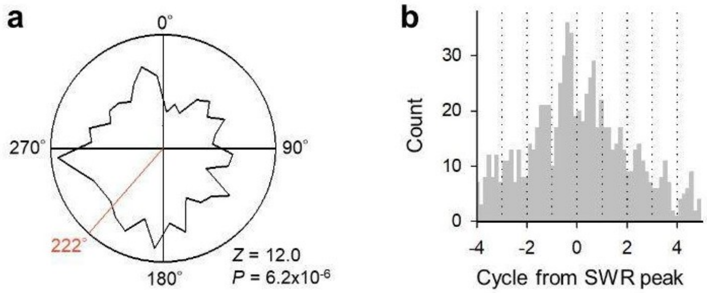

Supplementary Fig. 1. Spike times are entrained by ripple oscillations. (**a**) The phase distribution of spikes against ripple oscillations. The mean angle is shown in red. Spikes were phase-locked to ripples. $Z = 12.0$, $P = 6.3 \times 10^{-6}$, *Rayleigh* test, $n = 1093$ spikes from 50 cells. (**b**) The cycle-by-cycle distribution of spike times relative to SWR peaks. The dotted lines divide cycles. Source data is provided as a Source Data file.

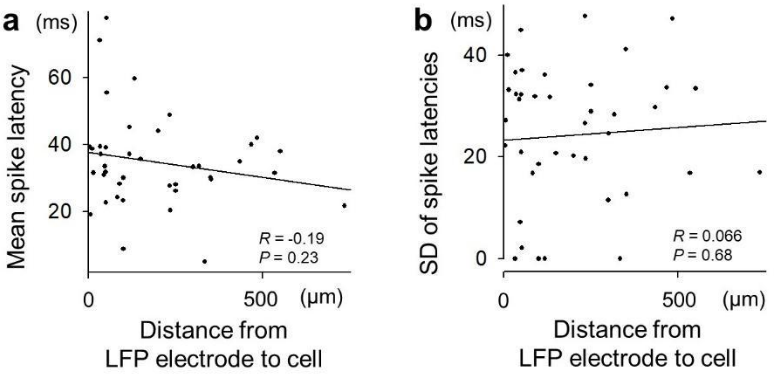

Supplementary Fig. 2. SWR-relevant spike times vary independently of the distances from the LFP electrodes to the cells. (**a**) The mean spike times relative to the SWR onset times were not correlated with the distances from the LFP electrodes to the cells. Each dot indicates a single cell. The line of best fit was determined using the least-squares method for a total of 40 cells. Significance was determined using a *t*-test of the correlation coefficient (two-sided). (**b**) The same as (a) but for the SD of the spike times for individual cells. Source data is provided as a Source Data file.

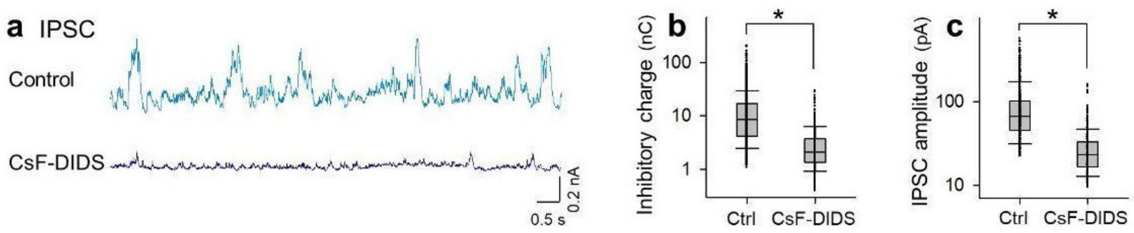

Supplementary Fig. 3. CsF-DIDS decreases IPSCs. (**a**) Representative traces of IPSCs in control and CsF-DIDS conditions. IPSCs were reduced by the intracellular application of CsF-DIDS. The holding potential was +10 mV as in Figure 2c. For CsF-DIDS conditions, the same intrapipette solution was used as in Figure 2b and 3a. (**b, c**) The inhibitory charges (b) and the IPSC amplitudes (c) were significantly reduced by CsF-DIDS. $P = 9.3 \times 10^{-59}$ and 6.2×10^{-109} for b and c respectively, Student's *t*-test (two-sided), $n = 912$ and 883 IPSCs for control and CsF-DIDS conditions, respectively. Box plots indicate the median and 25–75% interquartile ranges, and whiskers cover 10−90% quantiles. Source data is provided as a Source Data file.

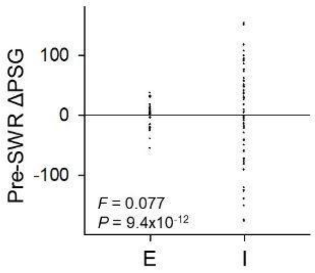

Supplementary Fig. 4. IPSGs are more variable prior to SWRs than EPSGs. ΔPSG values were averaged for EPSG (E) and IPSG (I) during pre-SWR periods and plotted after subtracting the means in each cell. ΔIPSGs were more variable than ΔEPSGs. $F = 0.077$, $P = 9.4 \times 10^{-12}$ (two-sided), $n = 34$, 57 SWRs from 5 cells for EPSG and IPSG, respectively. Source data is provided as a Source Data file.

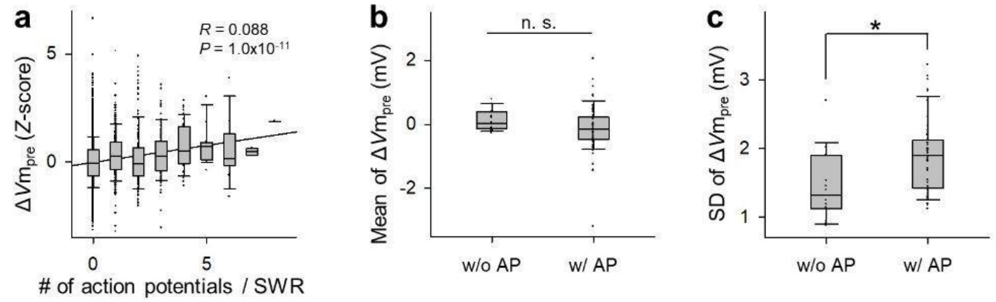

Supplementary Fig. 5. Larger pre-SWR hyperpolarizations are associated with lower action potential probabilities during SWRs. (**a**) The $\Delta V\mathrm{m}_{\mathrm{pre}}$ values (*Z*-scored in each cell) were positively correlated with the numbers of action potentials per SWR. $R = 0.088$, $P = 1.0 \times 10^{-11}$, *t*-test of the correlation coefficients (two-sided), $n = 5{,}971$ SWR events. (**b**) The means of $\Delta V\mathrm{m}_{\mathrm{pre}}$ values of the neurons with and without action potentials during SWRs were not significantly different. $P = 0.22$, $t_{62} = 1.2$, Student's *t*-test (two-sided), $n = 14$ inactive and 50 active cells. (**c**) The standard deviations of $\Delta V\mathrm{m}_{\mathrm{pre}}$ values of active neurons that showed at least one action potential during SWRs were significantly higher than those of inactive neurons during SWRs. $P = 0.0077$, $t_{62} = -2.8$, Student's *t*-test (two-sided), $n = 14$ inactive and 50 active cells. All box plots in this figure indicate median and 25–75% interquartile ranges; whiskers cover 10−90% quantiles. Source data is provided as a Source Data file.

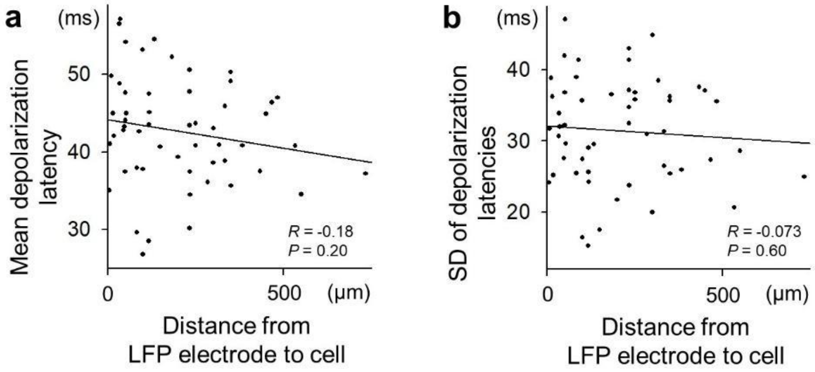

Supplementary Fig. 6. SWR-relevant depolarization peak times vary independently of the distances from the LFP electrodes to the cells. (**a**) The mean depolarization peak times relative to the SWR onset times were not correlated with the distances from the LFP electrodes to the cells. Each dot indicates a single cell. The line of best fit was determined using the least-squares method for a total of 54 cells. Significance was determined using a *t*-test of the correlation coefficient (two-sided). (**b**) The same as (a) but for the SD of the depolarization peak times for individual cells. Source data is provided as a Source Data file.

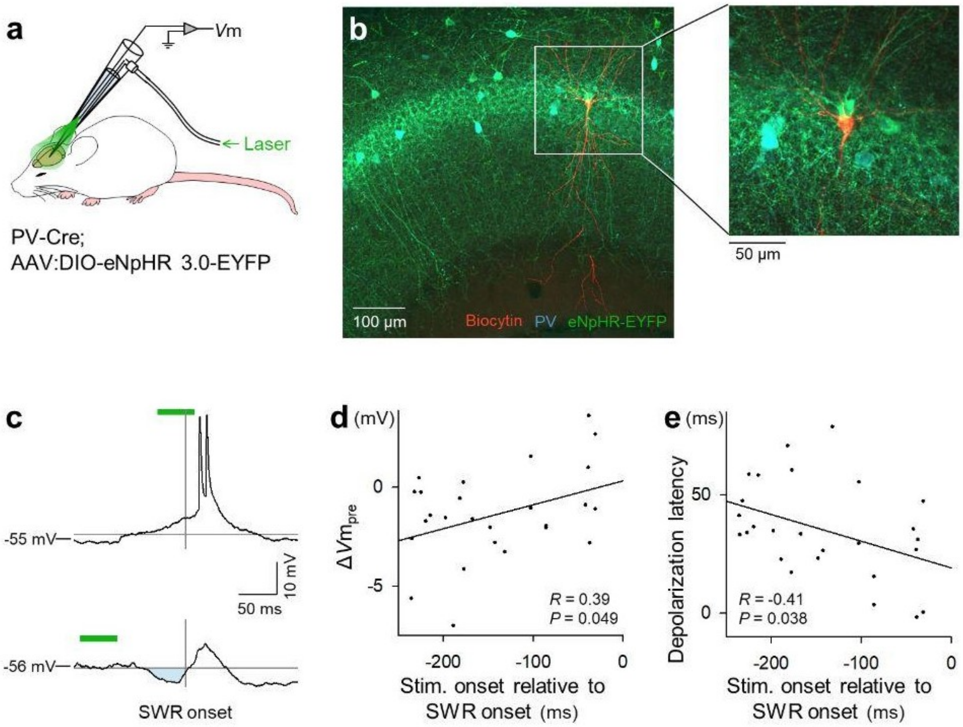

Supplementary Fig. 7. Optogenetic pre-SWR inhibition of PV-positive interneurons advances the SWR-relevant depolarization peak times of pyramidal cells. (**a**) Schematic description of optogenetic inhibition of the activity of PV-positive interneurons simultaneously with *in vivo* whole-cell recordings from single CA1 pyramidal neurons. (**b**) *Post hoc* biocytin-based visualization of a recorded pyramidal neuron (red) and confirmation of the expression of eNpHR3.0-EYFP (green) in PV-immunoreactive cells (blue). The same results were obtained for all 10 cells in 7 mice. (**c**) Representative SWR-relevant *V*m traces of CA1 pyramidal cells with green light stimulation, shown by green bars, within (*top*) and out of (*bottom*) the pre-SWR periods. The light blue area shows the pre-SWR hyperpolarization. (**d**) The relationship between $\Delta V\mathrm{m}_{pre}$ and the timings of stimulation onsets relative to the subsequent SWR onsets. (**e**) The relationship between the depolarization peak times and the timings of stimulation onsets relative to the subsequent SWR onsets. For (d) and (e), the line of best fit was determined using the least-squares method for a total of 26 SWRs in all 10 cells from 7 mice, and significance was determined using a *t*-test of the correlation coefficient (two-sided). Source data is provided as a Source Data file.

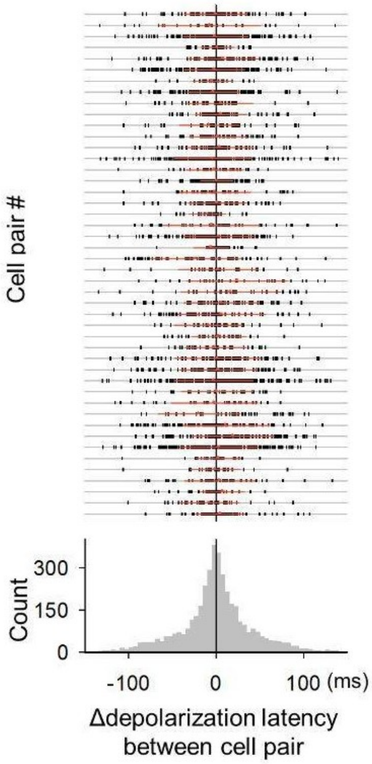

Supplementary Fig. 8. There is no tendency for one cell to depolarize earlier than another during SWRs. *Top*: Time differences in the depolarization peaks between all 46 cell pairs are shown in a raster plot, in which the pair order is sorted so that the distances between the cell pairs are shorter in higher rows. The six cases where we could not measure the distances are shown in the six bottom rows. In each cell pair, the cell located more medially in the hippocampus was used as a reference to calculate the time difference. Each vertical tick mark indicates a single SWR event. Red dots and lines are the means ± SDs of the time differences in the corresponding cell pairs. *Bottom*: Distribution of the depolarization peak time differences of all 4,039 SWRs in a total of 46 cell pairs. Source data is provided as a Source Data file.

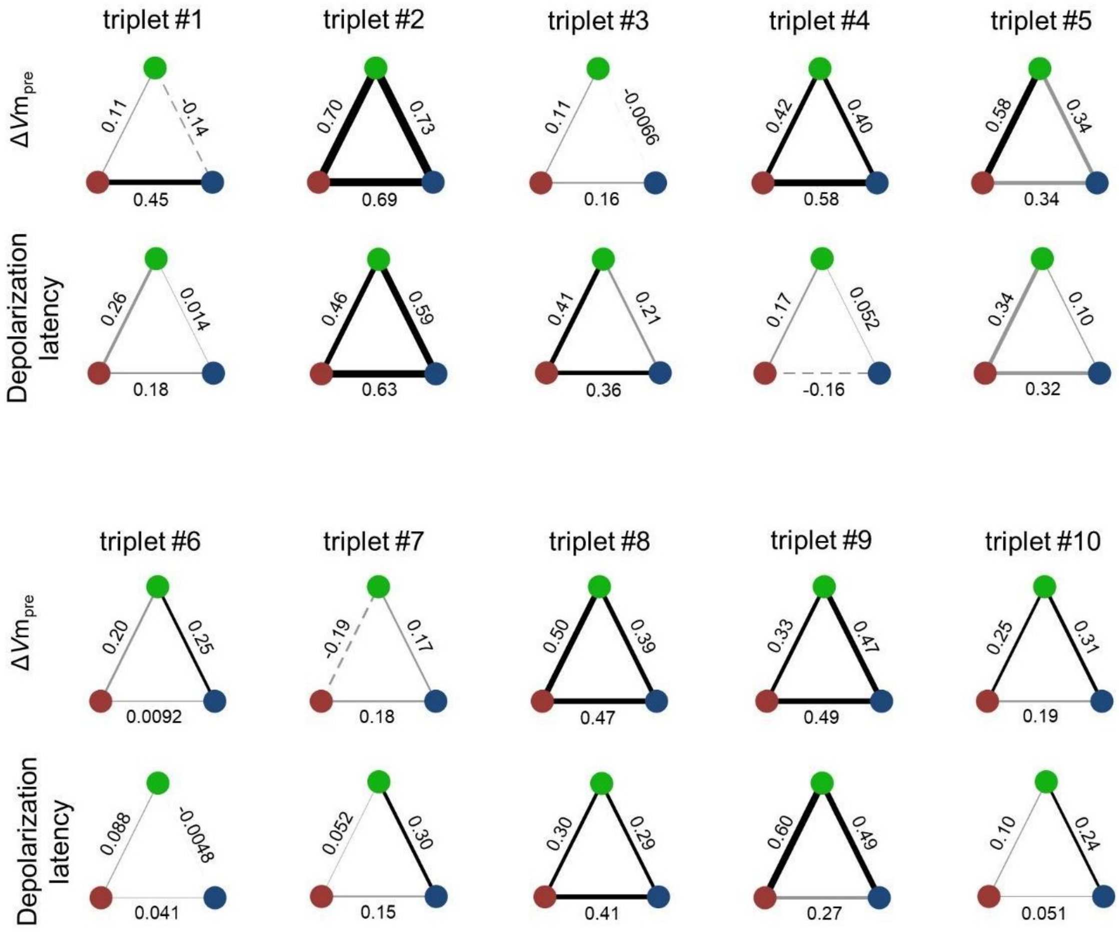

Supplementary Fig. 9. Correlation coefficients of $\Delta V\mathrm{m}_{pre}$ and depolarization peak times between CA1 neurons. The same analyses as in Figure 6a were conducted for $\Delta V\mathrm{m}_{pre}$ (top row in each pair) and the depolarization peak times (bottom row in each pair) in all 10 datasets of triplet patch-clamp recordings. Each circle represents a single CA1 neuron, and each triangle indicates three simultaneously patched cells. The line thickness corresponds to the correlation coefficient, which is also inscribed along the line. Lines are shown in black when the correlation coefficients were significant according to a *t*-test (two-sided); otherwise, they are shown in gray. Dashed lines indicate negative correlation coefficients, whereas continuous lines show positive correlation coefficients, with thicker lines indicating larger values.

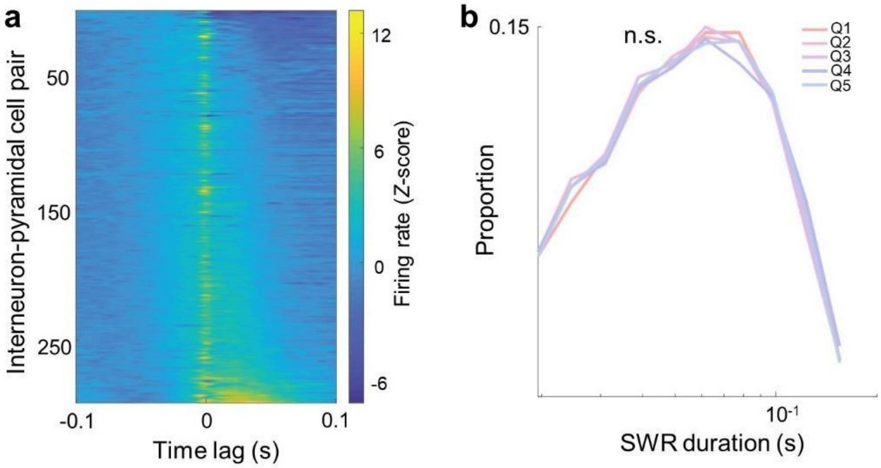

Supplementary Fig. 10. Inhibition does not affect SWR duration. (a) Cross-correlograms between interneurons and pyramidal cells with significantly delayed spikes across quintiles defined by pre-SWR firing rates of the interneurons. Cross-correlograms were computed outside of SWRs. (**b**) Distribution of SWR durations across quintiles. Increased pre-SWR firing rates of interneurons were not associated with changes in SWR duration (two-sided).

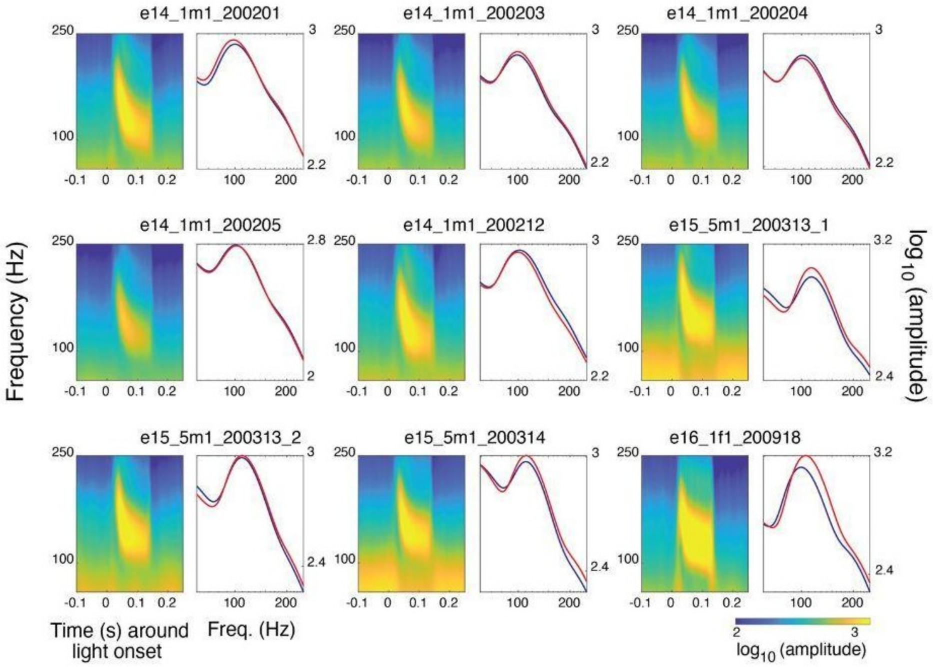

Supplementary Fig. 11. All light intensities induce SWRs. Average light onset triggered wavelet spectrograms of all 9 recording sessions in which pre-SWR firing interneurons were identified. The light-pulse power spectra, averaged across pulses, are shown to the right of each wavelet spectrogram. The blue/red line shows the average across the lowest/highest light intensity pulses, showing that high frequency oscillations did not depend on light intensities used in these experiments.
