# Supplementary material for: Inhibition allocates spikes during hippocampal ripples
Source: Nat Commun. 2022 Mar 11;13:1280. doi: 10.1038/s41467-022-28890-9 (PMC8917132; doi:10.1038/s41467-022-28890-9)
Supplement: Supplementary file 3 — Reporting Summary [file 41467_2022_28890_MOESM3_ESM.pdf]

nature portfolio

Corresponding author(s): Yuji Ikegaya
György Buzsáki

Last updated by author(s): Jan 28, 2022

Reporting Summary

Nature Portfolio wishes to improve the reproducibility of the work that we publish. This form provides structure for consistency and transparency in reporting. For further information on Nature Portfolio policies, see our Editorial Policies and the Editorial Policy Checklist.

Please do not complete any field with "not applicable" or n/a. Refer to the help text for what text to use if an item is not relevant to your study.
For final submission: please carefully check your responses for accuracy; you will not be able to make changes later.

Statistics

For all statistical analyses, confirm that the following items are present in the figure legend, table legend, main text, or Methods section.

n/a	Confirmed	
☐	☒	The exact sample size (*n*) for each experimental group/condition, given as a discrete number and unit of measurement
☐	☒	A statement on whether measurements were taken from distinct samples or whether the same sample was measured repeatedly
☐	☒	The statistical test(s) used AND whether they are one- or two-sided<br>*Only common tests should be described solely by name; describe more complex techniques in the Methods section.*
☐	☒	A description of all covariates tested
☐	☒	A description of any assumptions or corrections, such as tests of normality and adjustment for multiple comparisons
☐	☒	A full description of the statistical parameters including central tendency (e.g. means) or other basic estimates (e.g. regression coefficient) AND variation (e.g. standard deviation) or associated estimates of uncertainty (e.g. confidence intervals)
☐	☒	For null hypothesis testing, the test statistic (e.g. F, t, r) with confidence intervals, effect sizes, degrees of freedom and P value noted<br>*Give P values as exact values whenever suitable.*
☐	☒	For Bayesian analysis, information on the choice of priors and Markov chain Monte Carlo settings
☒	☐	For hierarchical and complex designs, identification of the appropriate level for tests and full reporting of outcomes
☐	☒	Estimates of effect sizes (e.g. Cohen's d, Pearson's r), indicating how they were calculated

Our web collection on statistics for biologists contains articles on many of the points above.

Software and code

Policy information about availability of computer code

Data collection	All patch-clamp recording data was acquired with pClamp 10.3 (Molecular Devices), an open source software.<br>Silicon probes from Cambridge NeuroTech (ASSY-156-E-1) were used for extracellular recordings. Unit recordings were conducted using the Intan RHD2132/RHD2000 interface board. Light pulse waveforms for in vivo optogenetics were generated using the Cyclops library available for Arduino/Teensy (https://github.com/jonnew/cyclops).
Data analysis	Automated spike sorting was performed using Kilosort1, followed by manual curation in Phy2. All offline computational analyses were performed using MATLABR2017b and Python3. Most custom code for analysis of extracellular unit data is available at https://github.com/buzsakilab/buzcode. Each analysis procedure is described in necessary detail in the Method section for others to execute. Any analysis codes are available by sending a request to the corresponding author.

For manuscripts utilizing custom algorithms or software that are central to the research but not yet described in published literature, software must be made available to editors and reviewers. We strongly encourage code deposition in a community repository (e.g. GitHub). See the Nature Portfolio guidelines for submitting code & software for further information.

Data

Policy information about availability of data

All manuscripts must include a data availability statement. This statement should provide the following information, where applicable:

- Accession codes, unique identifiers, or web links for publicly available datasets
- A description of any restrictions on data availability
- For clinical datasets or third party data, please ensure that the statement adheres to our policy

The data that support the findings of the study are available from the corresponding author upon reasonable request.

Field-specific reporting

Please select the one below that is the best fit for your research. If you are not sure, read the appropriate sections before making your selection.

☒ Life sciences ☐ Behavioural & social sciences ☐ Ecological, evolutionary & environmental sciences

For a reference copy of the document with all sections, see nature.com/documents/nr-reporting-summary-flat.pdf

Life sciences study design

All studies must disclose on these points even when the disclosure is negative.

Sample size	For patch-clamp recordings, sample size (the number of triple patching datasets and single whole-cell recordings in awake condition and from PV-Cre mice) was determined by previous publications that performed in vivo whole-cell recordings from the hippocampus (Gan et al., Neuron, 2017). Single and dual whole-cell recordings in anesthetized condition were obtained more frequently in this order in the process of achieving triple whole-cell recordings, which resulted in larger number of data than triple patching datasets (Jouhanneau et al., 2018, 2019).<br>For unit recordings, n=6 animals were used, exceeding the typical cohort sizes (3-4 animals) of studies examining SWRs (Foster and Wilson 2006; Grosmark and Buzsaki 2016). Our larger cohort allows for an examination of individual variability and accurate assessment of population averages.<br>No further statistical methods were used to predetermine the sample size.
Data exclusions	For patch-clamp recordings, data without enough quality (recordings with mean Vm less than -50 mV and action potentials below -20 mV) and enough number of SWRs (recordings with less than 30 SWR events) were excluded for reliable statistical analyses. The criteria is described in the Method section of the manuscript.<br>For extracellular unit recordings, one animal was excluded from further analysis for a lack of preSWR firing interneurons (Figure 7).
Replication	All conclusions of this study are based on recordings of populations of cells, which were consistent in all animals we used.
Randomization	We used a within subject shuffle to generate a null hypothesis (Figure 5b, 7g, 8g). Therefore, the same variability and autocorrelation structure present in each signal is preserved in our null distributions. For Figure 2b inset, which is the only figure with comparison between experimental groups, we randomly allocated mice into control and CsF-DIDS groups.
Blinding	No blinding was done in this study because knowledge of experimental conditions was required during data collection.

Reporting for specific materials, systems and methods

We require information from authors about some types of materials, experimental systems and methods used in many studies. Here, indicate whether each material, system or method listed is relevant to your study. If you are not sure if a list item applies to your research, read the appropriate section before selecting a response.

Materials & experimental systems

n/a	Involved in the study	
☐	☒	Antibodies
☒	☐	Eukaryotic cell lines
☒	☐	Palaeontology and archaeology
☐	☒	Animals and other organisms
☒	☐	Human research participants
☒	☐	Clinical data
☒	☐	Dual use research of concern

Methods

n/a	Involved in the study	
☒	☐	ChIP-seq
☒	☐	Flow cytometry
☒	☐	MRI-based neuroimaging

Antibodies

Antibodies used	In figure S6, we used the primary and secondary antibodies as follows; a chicken primary antibody against green fluorescent protein (GFP; 1:1000, ab13970, Abcam), a guinea pig primary antibody against parvalbumin (1:500, 195 004, Synaptic Systems), Alexa Fluor 488-conjugated goat secondary antibody against chicken IgG (1:500, A11039, Thermo Fisher Scientific), Alexa Fluor 594-conjugated goat secondary antibody against guinea pig IgG (1:500, A11076, Thermo Fisher Scientific).
Validation	Chicken anti-GFP was shown to react with YFP by the manufacturer. Guinea pig anti-PV was shown to stain parvalbumin in mice by the manufacturer. Alexa 488-labeled goat anti-chicken IgG and Alexa 594-labeled anti-guinea pig IgG were<br>shown to immunohistochemically stain the corresponding primary antibodies by the manufacture.

Animals and other organisms

Policy information about studies involving animals; ARRIVE guidelines recommended for reporting animal research

Laboratory animals	For patch-clamp recordings except for Figure S6, we used male ICR mice (28 to 45 days old). For optogenetic manipulation with

Laboratory animals	patch-clamp recordings in Figure S6, we used PV-Cre mice (4 male, 1 female; 5 to 8 weeks old).<br>For unit recordings, adult wild-type C57BL/6J mice (5 male, 1 female; 4 to 6 months old) were used.<br>All animals were housed under a 12/12-h light-dark cycle (light from 07:00 to 19:00) at 22 ± 1 °C with food and water provided ad libitum.
Wild animals	The study did not involve wild animals.
Field-collected samples	The study did not involve samples collected from the field.
Ethics oversight	For patch-clamp recordings:<br>Animal experiments were performed with the approval of the animal experiment ethics committee at the University of Tokyo (approval numbers: P29-9) and in accordance with the University of Tokyo guidelines for the care and use of laboratory animals. These experimental protocols were conducted in accordance with the Fundamental Guidelines for the Proper Conduct of Animal Experiments and Related Activities in Academic Research Institutions (Ministry of Education, Culture, Sports, Science and Technology, Notice No. 71 of 2006), the Standards for Breeding and Housing of and Pain Alleviation for Experimental Animals (Ministry of the Environment, Notice No. 88 of 2006) and the Guidelines on the Method of Animal Disposal (Prime Minister's Office, Notice No. 40 of 1995).<br><br>For unit recordings:<br>All experiments were conducted with the approval of the Institutional Animal Care and Use Committee of New York University. Medical Center.

Note that full information on the approval of the study protocol must also be provided in the manuscript.

This checklist template is licensed under a Creative Commons Attribution 4.0 International License, which permits use, sharing, adaptation, distribution and reproduction in any medium or format, as long as you give appropriate credit to the original author(s) and the source, provide a link to the Creative Commons license, and indicate if changes were made. The images or other third party material in this article are included in the article's Creative Commons license, unless indicated otherwise in a credit line to the material. If material is not included in the article's Creative Commons license and your intended use is not permitted by

statutory regulation or exceeds the permitted use, you will need to obtain permission directly from the copyright holder. To view a copy of this license, visit http://creativecommons.org/licenses/by/4.0/
